# Parental income and drug use disorder among second-generation individuals in Sweden: effect modification by immigrant background and region of origin

**DOI:** 10.1186/s12889-026-27931-y

**Published:** 2026-05-29

**Authors:** Kenta Okuyama, Sara Larsson Lönn, Ardavan M. Khoshnood, Shervin Assari, Jan Sundquist, Kristina Sundquist

**Affiliations:** 1 Center for Primary Health Care Research, Department of Clinical Sciences Malmö, Lund University, Jan Waldenströms gata 35, Malmö, Skåne 20502 Sweden; 2 University Clinic Primary Care, Skåne University Hospital, Region Skåne, Sweden; 3 Emergency Medicine, Department of Clinical Sciences Malmö, Lund University, Skåne University Hospital, Malmö, Sweden; 4 Department of Internal Medicine, Charles R Drew University of Medicine and Science, Los Angeles, CA USA; 5 Department of Family Medicine, Charles R Drew University of Medicine and Science, Los Angeles, CA USA; 6 Department of Urban Public Health, Charles R Drew University of Medicine and Science, Los Angeles, CA USA; 7 Marginalization Related Diminished Returns Research Center, Los Angeles, CA USA

**Keywords:** Diminished return, Drug use disorder, Effect modification, Parental income, Region of origin, Second generation

## Abstract

**Background:**

Evidence is limited for the association between parental income during childhood and adolescence and the subsequent risk of drug use disorder (DUD) in the second generation and whether the association is modified by region of origin. Identifying the association and effect modification by immigrant background and region of origin would provide an important knowledge base to consider tailored policies.

**Methods:**

We used nationwide longitudinal Swedish data on 1,137,721 non-immigrants and 293,636 s-generation individuals who turned 15 years of age between 2005 and 2020. Cox proportional hazard models were used to examine the associations between parental income and DUD by immigrant background and region of origin. Parental income was assessed in ranked percentiles based on family disposable income when the study individuals were 14 years of age. In addition, accumulated exposure and critical periods of low parental income were assessed at the ages 0–4, 5–9, and 10–14. DUD was assessed using medical/legal registers 2005–2020 when the study individuals were between 15 and 25 years old.

**Results:**

Higher parental income was associated with a decreased risk of DUD, while the association was weaker in the second generation, such as in those from Asia, Africa, and Middle East or North Africa (MENA) in males, and Latin America or Caribbean, Africa, and MENA in females. Accumulated exposure to low parental income was associated with an increased risk of DUD, while the association was weaker in the second generation, such as in those from MENA.

**Conclusions:**

Financial security in early life may lower the subsequent risk of DUD. However, the associations varied by immigrant background and region of origin.

**Supplementary Information:**

The online version contains supplementary material available at 10.1186/s12889-026-27931-y.

## Background

Drug use disorder (DUD) is highly prevalent globally during young adulthood [1, 2]. Although the overall prevalence of DUD in Sweden remains lower than in many other European countries, population surveys and studies suggest an increasing trend, with past-year illicit drug use among the general population rising from 2.5% in 2014 to approximately 3.5% in 2020 [3, 4]. Increases in drug-related harms, including drug-related mortality and treatment demand, have also been reported in recent years [5, 6].

While evidence remains inconclusive, individuals with immigrant background may be at an elevated risk of using drugs [7, 8]. The number of international migrants has markedly increased over the past 50 years [9]. In Europe, 22% of the population has been defined as being either first-generation immigrants (i.e., those who were born outside of receiving countries) or second-generation immigrants (i.e., those who were born in receiving countries to one or two foreign-born parents) [10]. In Sweden, one-third of the population have been classified as either first- or second-generation immigrants from diverse regions of the world [11]. While evidence is limited regarding the prevalence of DUD among these populations, the second generation is often exposed to stress in the family environment as well as the school or community environment, and a higher risk of substance use disorders in this group than in non-immigrants and first-generation immigrants has been suggested [12, 13]. Especially, those from geographically and culturally distant regions may experience greater stress, and that could elevate the risk of DUD further [14].

Parental income during childhood and adolescence has been reported to be an important predictor of substance use disorder in young adulthood [15–17]. Higher parental income is an important socioeconomic factor that could ensure stable family circumstances, well-functioning parent-child relationships, and access to educational resources, which, in turn, may lead to a decreased risk of DUD [16]. While the underlying mechanisms and the strength of the association with parental income may differ across types of drugs, e.g., opioids, cannabis, and stimulants, large cohort studies conducted in Nordic countries have consistently reported that higher parental income is associated with an overall decreased risk of DUD [15–17]. Evidence from the US suggests that the protective effects of parental socioeconomic status, e.g., parental education, on substance use among individuals with an immigrant background may be smaller, which is suggested to be related to marginalization-related diminished returns (MDRs) [18–20]. Potential MDRs of education and income on health outcomes have also been observed in Europe, and seem to be particularly pronounced among individuals from Africa, Asia, and Middle East or North Africa [21]. However, little is known about whether there is an association between parental income and DUD in the second generation in Europe, as well as in other regions of the world, and if the association is modified by immigrant background and region of origin, i.e., whether the protective effects of parental income are diminished among certain groups.

The second generation is, in general, more socioeconomically disadvantaged compared to non-immigrants (i.e., those who were born in the same country where their parents were born). Despite these disadvantages, previous research suggests that some second-generation groups may converge toward or even outperform non-immigrant peers in educational outcomes or aspirations in both the US and several European countries, although patterns vary substantially across groups [22–25]. On the other hand, many also tend to be more challenged in school and in the labor market in Europe, as well as in Sweden, and in turn, to a higher extent be exposed to disadvantaged socioeconomic circumstances [22, 26]. While one large cohort study in Sweden has reported that accumulated exposure to low parental income during childhood was associated with an increased risk of DUD in adulthood [17], no research studies have investigated the accumulated exposure of low parental income for DUD risk in the second generation. In addition, there is limited knowledge about the critical period during childhood and adolescence (e.g., 0–4, 5–9, and 10–14 years old) concerning exposure to low parental income for DUD risk, i.e., in which age periods parental income and DUD are strongest associated [27]. Indications regarding the most critical periods could be useful to consider targeted policies and interventions [28].

Given this background, our study aimed to investigate whether parental income is associated with the risk of DUD and if the association is modified by immigrant background and region of origin. Furthermore, we aimed to investigate the accumulated exposure and critical periods of low parental income for DUD, and whether the associations are modified by immigrant background and region of origin. Since the outcome defines DUD as a composite measure, the findings should be interpreted in light of potential, unmeasured heterogeneity across types of drugs.

## Methods

### Data

We used nationwide longitudinal population register-based data derived in Sweden. These register-based data were linked by pseudonymized serial numbers, which replaced national identification numbers to ensure the integrity of study individuals. Detailed information on register data used in this study can be found in the Supplementary material (eTable 1).

### Study population

We included individuals who turned 15 years of age during the period 2005 and 2020. The study individuals were categorized as follows: Non-immigrants - if they were born in Sweden to two Swedish-born parents; Second-generation individuals - if they were born in Sweden to at least one foreign-born parent, which is consistent with other previous studies [29, 30]. The individuals with DUD at the age of 15 were excluded (which represented 2% of those who were registered for DUD between the ages of 15 and 25). The study individuals were followed until the first event of DUD, death, emigration, age 25, or the end of the study period (2020), whichever came first. The flowchart of our study population can be found in the Supplement (eFigure 1), and our final study population included 1,137,721 non-immigrants and 293,636 s-generation individuals.

### Outcome

DUD was assessed by WHO’s International Classification of Diseases (ICD) codes of Inpatient and Outpatient records in the National Patient Register, as well as suspicion and convictions (in lower courts) from the Crime Register, respectively. The definition of DUD is in line with previous studies [31, 32] and detailed information can be found in the Supplementary material (eTable 2).

### Exposure

Parental income was assessed by total household family disposable income, which is defined as total income including salary from work, capital, social service, and all other allowances, minus taxes paid based on data from the longitudinal integrated database for health insurance and labour market studies (LISA); this definition has been widely used in register-based studies [33]. Family disposable income at the age of 14 was grouped into 100 percentiles, and the lowest 5% income group was excluded, as those low incomes may not represent living in representative low-income households (Supplement eFigure 2). To investigate the accumulated exposure and critical periods of low parental income, below 60% of the median family disposable income was used as a threshold [17]. We did not equivalize income for household size and composition because detailed and consistent household composition data were not available throughout the entire study period. Therefore, our measure was able to capture the total socioeconomic resources in a family, i.e., the potentially broader material environment in which children were raised, rather than per capita allocation. The exposure to low parental income was assessed during three periods, which is in line with previous studies; 0–4, 5–9, and 10–14 years of age, and treated as an ordered categorical variable to investigate the accumulated exposure, i.e., (1) not exposed, (2) exposed during one period, (3) exposed during two periods and (4) exposed during three periods [34]. To investigate critical periods of low parental income, the exposure to low parental income was grouped into eight categories (Supplement eFigure 4). In supplementary analyses, the social welfare assistance-receiving status of parents was used as an alternative measure of low parental income.

### Effect modifier

We examined whether immigrant background and region of origin could modify the effects of parental income. Immigrant background was divided into non-immigrants and second-generation individuals from six regions based on the country of birth of parents, i.e., Africa, Asia, Eastern Europe, Latin America or Caribbean (below referred to as Latin America), Middle East or North Africa (MENA), and Western regions (Supplement eTable 3) [35, 36].

### Covariates

Birth year, parental education, and parental DUD were considered as potential confounders. Parental education was assessed from the LISA and categorized into low (compulsory school), middle (high school), and high (university or higher education). Parental DUD was assessed from Inpatient and Outpatient records in the National Patient Register, and suspicion and convictions in the Crime Register. Highest parental education and parental DUD were assessed at any time during the study period. Parental DUD onset at a later age was rare, and we therefore assumed that the results would not be affected by later registrations.

### Statistical analysis

We used Cox proportional hazard models with time to the first event of DUD as the outcome. The association between percentile-ranked parental income and DUD and the effect modification by immigrant background and region of origin was examined by including the interaction terms of parental income and immigrant background, and parental income and region of origin, adjusting for potential confounders. We explored the adequacy of the linear specification by comparing it with more flexible functional forms, including spline and quadratic terms. These analyses did not indicate meaningful deviations from linearity, and the linear specification provided a more interpretable model for addressing our research questions, particularly when examining differences across population groups. We present the results as hazard ratios (HR) with a 95% confidence interval (CI). For the effect modification, we present the derived association for each group.

To investigate the association between accumulated exposure to low parental income and DUD, HR and 95% CI of DUD were estimated by accumulated exposure categories (not exposed to any periods as a reference). To investigate the critical periods of exposure to low parental income, HR and 95% CI of DUD were estimated by eight categories of exposure (not exposed to any critical periods as a reference). The effect modification by immigrant background and region of origin was assessed by including the interaction terms of immigrant background and region of origin for the analysis of accumulated exposure and critical periods, respectively. We examined critical periods by comparing the magnitude of the estimated HRs of different age periods, Akaike Information Criterion (AIC), and likelihood ratio tests. As supplementary analyses, the analyses for accumulated exposure and critical periods were repeated with the status of receiving social welfare assistance, instead of below 60% of the median parental income.

To assess the proportional hazards assumption, we used graphical diagnostics to evaluate the Schoenfeld residuals and judged that the assumption was met. As the incidence and mechanisms of DUD may differ by sex, all analyses were stratified by the study individuals’ sex [37]. All analyses were made in RStudio 2024.12.0 [38].

## Results

Table 1 shows the characteristics of the study individuals by immigrant background. In the second generation, the mean parental income was lower, and the proportion of low parental education and parental DUD was higher compared to non-immigrants. The proportion of region of origin was highest in the Western region, followed by MENA, Eastern Europe, Asia, Africa, and Latin America.

**Table 1:** Descriptive statistics of characteristics for study individuals

	Non-immigrants	Second generation
Total		
N (%)	1,137,721 (79.49%)	293,636 (20.51%)
Males		
N (%)	585,004 (79.59%)	150,033 (20.41%)
Drug use disorder		
N (%)	43,364 (7.41%)	19,929 (13.28%)
Parental income		
Mean (SD)	64.92 (4.43)	55.07 (23.76)
Birth year		
Mean (SD)	1996.52 (4.44)	1997.01 (4.39)
Parental education, N (%)		
Low	10,219 (1.75%)	9,852 (6.57%)
Middle	129,066 (22.06%)	34,096 (22.73%)
High	445,719 (76.19%)	106,085 (70.71%)
Parental drug use disorder		
N (%)	27,813 (4.75%)	12,212 (8.14%)
Region of origin, N (%)		
Africa	-	10,320 (6.88%)
Asia	-	22,634 (15.09%)
Eastern Europe	-	27,137 (18.09%)
Latin America or Caribbean	-	10,057 (6.7%)
Middle East or North Africa	-	31,052 (20.7%)
West	-	48,833 (32.55%)
Females		
N (%)	552,717 (79.38%)	143,603 (20.62%)
Drug use disorder		
N (%)	15,495 (2.8%)	5,557 (3.87%)
Parental income		
Mean (SD)	64.89 (22.44)	54.96 (23.72)
Birth year		
Mean (SD)	1996.53 (4.44)	1997.03 (4.38)
Parental education, N (%)		
Low	9,822 (1.78%)	9,300 (6.48%)
Middle	121,791 (22.03%)	33,072 (23.03%)
High	421,104 (76.19%)	101,231 (70.49%)
Parental drug use disorder		
N (%)	26,505 (4.8%)	11,791 (8.21%)
Region of origin, N (%)		
Africa	-	10,107 (7.04%)
Asia	-	21,530 (14.99%)
Eastern Europe	-	26,101 (18.18%)
Latin America or Caribbean	-	9,505 (6.62%)
Middle East or North Africa	-	29,902 (20.82%)
West	-	46,458 (32.35%)

Table 2 shows the incidence rates (IRs) of DUD per 100,000 person-years by immigrant background and region of origin. Among males, IRs were higher in all second-generation groups compared to non-immigrants, especially among those with African origin and MENA. Among females, IRs were also higher in all second-generation groups compared to non-immigrants, especially among those with origin of Africa and Latin America. Overall, IRs of DUD were higher among males than females, and the differences in IRs between non-immigrants and second-generation individuals were greater among males than females.

**Table 2:** Incidence rate of drug use disorder per 100,000 person-years by immigrant background and region of origin

	Males	Females
	IR (95% CI)	IR (95% CI)
Non-immigrants	1189 (1178; 1201)	437 (430; 444)
Second generation		
Africa	4152 (3980; 4330)	765 (696; 840)
Asia	1865 (1790; 1941)	477 (440; 517)
Eastern Europe	2187 (2114; 2263)	587 (550; 626)
Latin America	2776 (2638; 2920)	950 (871; 1034)
MENA	3205 (3119; 3292)	620 (584; 658)
West	1732 (1686; 1779)	658 (630; 687)
*IR* Incidence rate, *CI* Confidence interval, *Latin America* Latin America or Caribbean, *MENA* Middle East or North Africa		

**Fig. 1:**
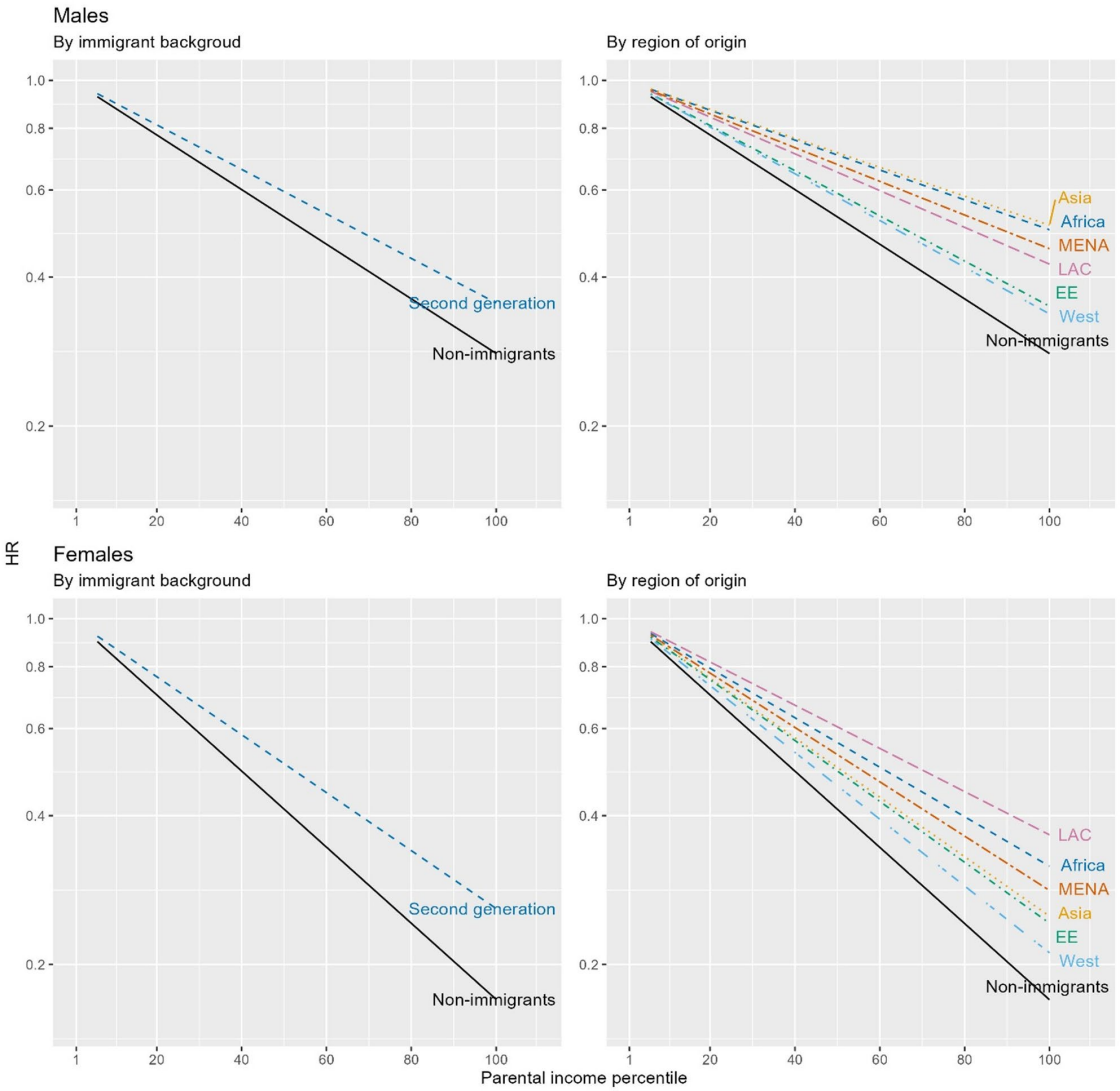
Hazard ratio (HR) of drug use disorder by parental income according to immigrant background and region of origin. Footnotes: HRs were derived from the Cox proportional hazard models adjusting for birth year, parental education, and parental drug use disorders. X-axis is 1-100 percentiles of parental income. Y-axis is in log-scale. EE: Eastern Europe. LAC: Latin America or Caribbean. MENA: Middle East or North Africa

Figure 1 shows the association between parental income and DUD according to immigrant background and region of origin. For all male and female non-immigrants and second-generation individuals, every percentile increase in parental income was associated with a decreased hazard of DUD. The association between parental income and DUD was weaker among all male and female second-generation groups, which is represented by the flatter slope of predicted lines in Fig. 1. Among males, the weakened association was most pronounced in the second generation from Asia, Africa, and MENA. Among females, the weakened association was most pronounced in the second generation from Latin America, Africa, and MENA. HRs and 95% CIs of DUD by ten-percentile increase in parental income by immigrant background and region of origin are presented in the Supplementary material (eTables 4 and 5). For example, by region of origin, HRs (95% CIs) were as follows for males: non-immigrants: 0.88 (0.88; 089), second generation from Africa: 0.93 (0.91;0.95), second generation from Asia: 0.93 (0.92;0.95), and second generation from MENA: 0.92 (0.91; 0.94). For females, the corresponding HRs (95% CIs) were as follows: non-immigrants: 0.84 (0.83; 0.84), second generation from Africa: 0.89 (0.85; 0.93), second generation from Latin America: 0.90 (0.87; 0.94), and second generation from MENA: 0.88 (0.86; 0.91) (eTable 5). The interaction terms were all statistically significant (results not shown), thus suggesting a significant effect modification by immigrant background and region of origin.

**Fig. 2:**
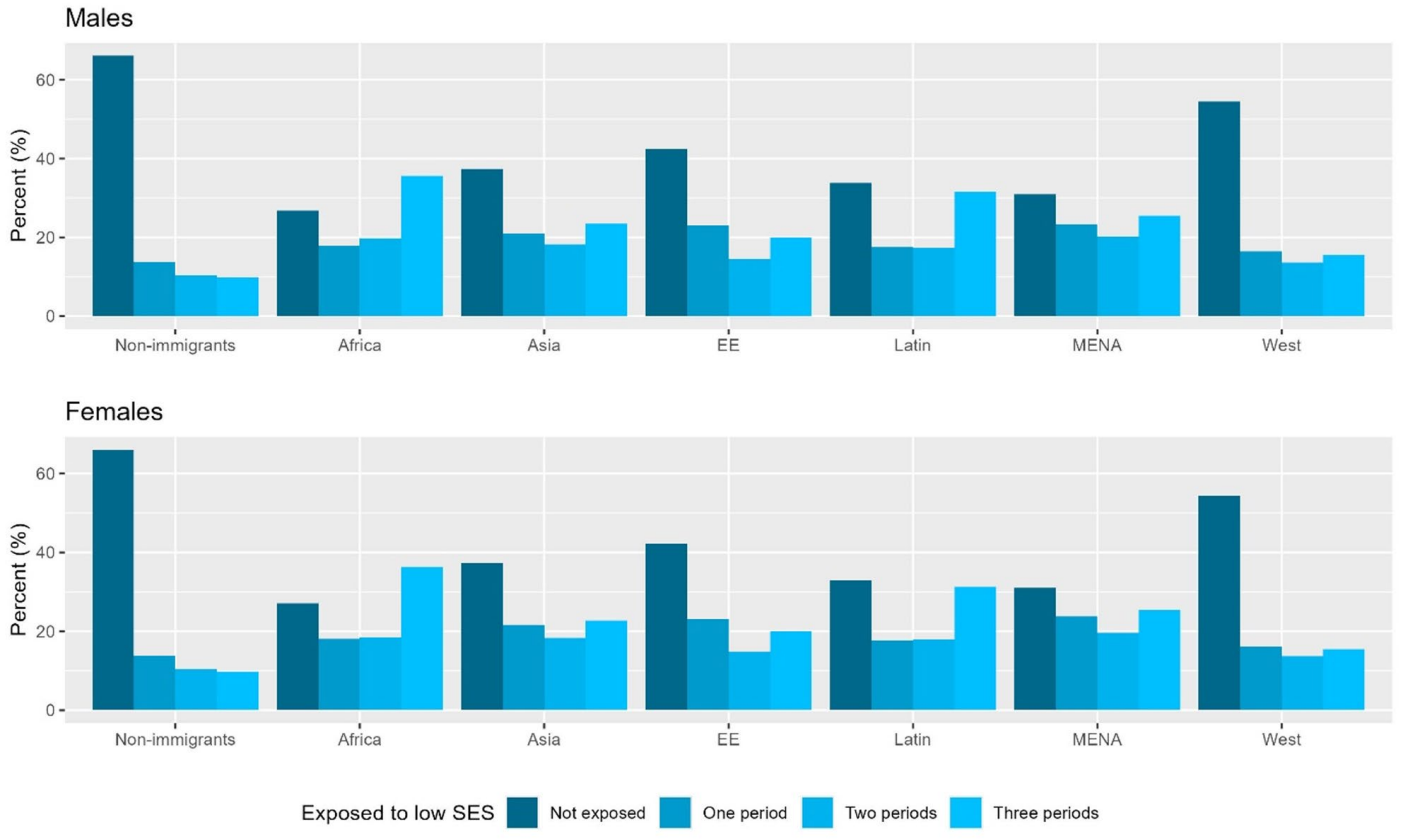
Proportion of individuals exposed to low parental income (i.e., less than 60% median parental income) at multiple age periods according to immigrant background and region of origin. Footnotes: EE: Eastern Europe. Latin: Latin America or Caribbean. MENA: Middle East or North Africa

Figure 2 shows that the proportion of individuals exposed to low parental income at multiple age periods was particularly high in the second generation from Africa, Latin America, and MENA, compared with non-immigrants, for both males and females.

**Fig. 3:**
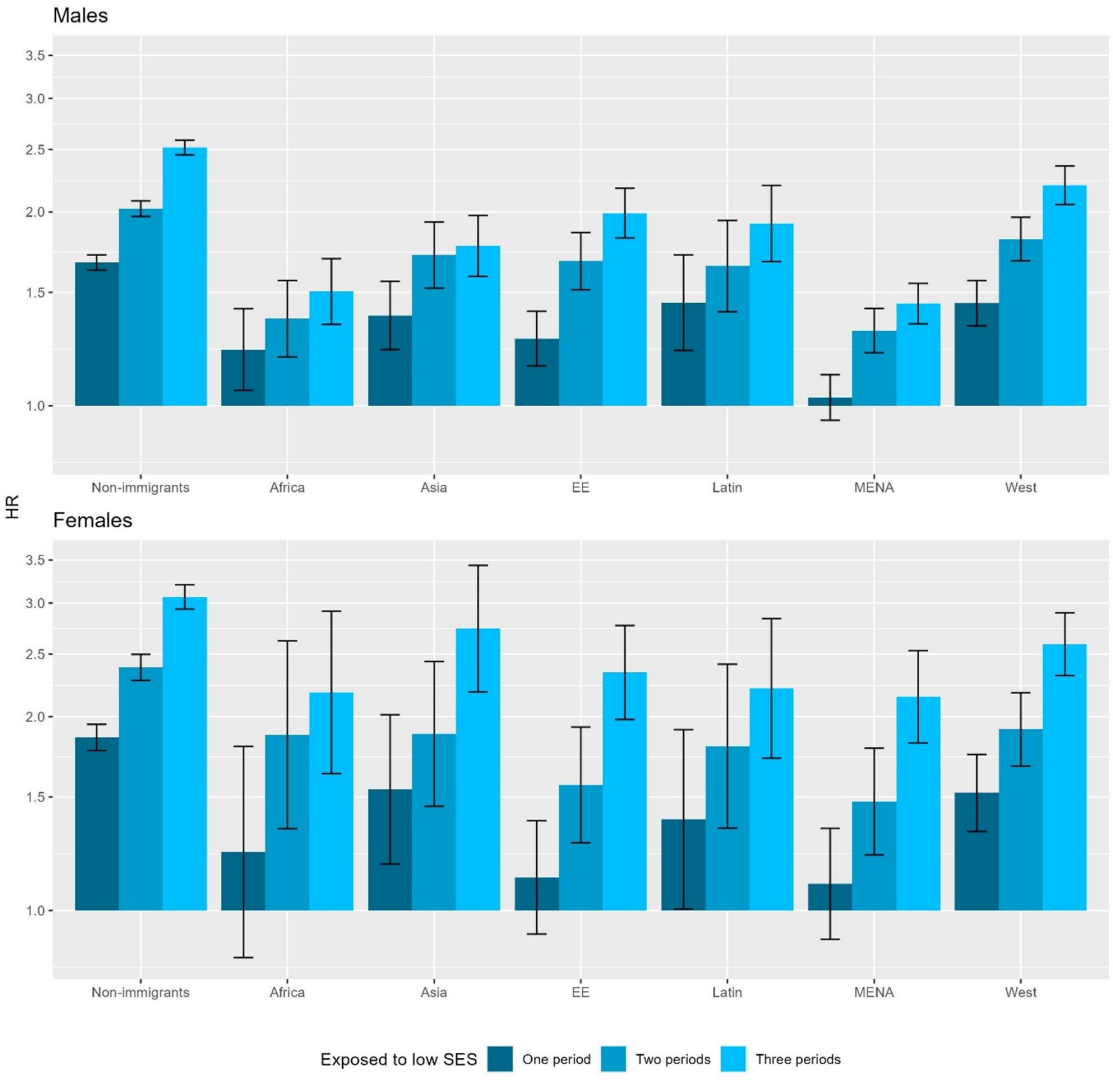
Hazard ratio (HR) of drug use disorder by accumulated exposure to low parental income (i.e., less than 60% median parental income) according to immigrant background and region of origin. Footnotes: HRs were derived from the Cox proportional hazard models adjusting for birth year, parental education, and parental drug use disorders. Y-axis is in log-scale. Not exposed to any periods is the reference. EE: Eastern Europe. Latin: Latin America or Caribbean. MENA: Middle East or North Africa

Figure 3 shows the association between accumulated exposure to low parental income (i.e., less than 60% median parental income) and DUD according to immigrant background and region of origin. For all male and female non-immigrants and second-generation individuals, accumulated exposure to low parental income was associated with an increased hazard of DUD. The associations were weaker among all male and female second-generation groups, represented by the shorter height of bars in Fig. 3. The weaker association was most pronounced in the second generation from MENA among both males and females. When social welfare receipt was alternatively used as the measure of accumulated exposure to low parental income in supplementary analyses, similar associations were observed (Supplement eFigure 2).

There was an indication that the exposure of low parental income for the period 10–14 years was more critical than other age periods for all male and female non-immigrants and second-generation individuals by comparing the HRs of different age periods (Supplement eFigure 3). This is partly supported by AIC and likelihood ratio tests, indicating that the models treating the age periods as eight rather than four categories were a better fit for the data. However, the association with accumulated exposure was more obvious for all male and female non-immigrants and second-generation individuals, i.e., the HRs of DUD increased with more periods of exposure to low parental income, regardless of specific age periods.

## Discussion

Our study indicated a significant association between parental income and DUD among non-immigrants and second-generation individuals, and the association was modified by immigrant background and region of origin. Specifically, the estimated protective effect of high parental income was smaller in the second generation, and that was more pronounced in those with the origin of Asia, Africa, and MENA among males, and Latin America, Africa, and MENA among females. The mechanisms between these patterns, being more pronounced in the second generation with non-European backgrounds, were not possible to examine in the present study but some underlying mechanisms behind the diminished returns of parental income may be linked to acculturative stress, as well as structural and social barriers that could persist despite socioeconomic advantage. Our study also suggested that the accumulated exposure to low parental income was associated with an increased risk of DUD among non-immigrants and second-generation individuals, but these associations were weaker in the second generation, particularly in those from MENA, for both males and females. There was also an indication that exposure to low parental income during the period 10–14 years old was more critical than other age periods for all male and female non-immigrants and second-generation groups.

The association between parental income and DUD corroborates previous studies [15, 16]. High parental income during childhood and adolescence is an important socioeconomic factor which could lead to a decreased risk of DUD [16]. While the association in our study indicates that the increase in parental income would be associated with a decreased risk of DUD among both non-immigrants and second-generation individuals, the potential effects may be smaller in the second generation. This is in line with several previous studies that identified MDRs, i.e., smaller protective effects of parental education and income on substance use among those with an immigrant background compared to non-immigrants [19, 20, 39, 40]. One potential explanation proposed in prior research is that socioeconomic resources may confer fewer health benefits among minority groups due to exposure to discrimination and structural barriers, which may be particularly relevant for certain minority populations in European contexts, such as those from non-European countries [21, 39]. There is also some evidence suggesting that the effects of income, mediated by financial stress, on brain structure and development, which are important predictors of DUD risk, may be different in individuals from minority backgrounds [41, 42].

While most studies that identified MDRs are from the US, several studies conducted in Europe have reported a weaker association between socioeconomic status and mental health outcomes among those with and immigrant background compared to non-immigrants [43, 44]. One of the novel findings from our study was that the potentially diminished protective effects were found specifically for parental income on DUD in the second generation, and that was more pronounced in those from certain regions, such as Africa, Asia, Latin America, and MENA. This is aligned with one of the recent studies of MDRs in European countries that observed the most pronounced diminished returns of income on health outcomes among individuals with origin from Africa, as well as Asia, and MENA [21]. These non-European minority groups may face structural inequalities ascribed to stigma and discrimination to a greater extent, which could diminish the protective effects of income [45]. In Sweden, despite long-term ambitions for equality in the labor market and in school, gaps exist in employment, wages, and school performance between those with and immigrant background and non-immigrants [46–49]. Previous studies suggest that non-European and non-Western migrants in Sweden, especially those from Africa, Asia, and Latin America, experience higher unemployment rates and lower income levels compared to non-immigrants [46, 47]. In addition, minority individuals often tend to segregate in deprived neighborhoods [50, 51], and these inequalities might diminish the effect of parental income on DUD. This is further supported by findings from the US that minority individuals with high income remain living in at-risk neighborhoods [52], which could diminish the protective effect of higher income on substance use [40]. Some of these mechanisms could potentially be examined using additional covariates available in register data, such as neighborhood deprivation, school characteristics, or parental labor market attachment. However, such studies were beyond the scope of the current one and warrant further investigation.

An additional notable pattern in our results is that the potential inequalities between non-immigrants and second-generation groups appeared to widen at higher levels of parental income, suggesting greater relative disparities at the upper end of the income distribution. This pattern is consistent with what has been described as an “integration paradox”; socioeconomic advancement does not necessarily translate into equivalent health gains among minority populations [18, 53]. One possible explanation is that upwardly mobile individuals from minority backgrounds may encounter persistent structural barriers, discrimination, or social exclusion that become more salient in higher socioeconomic contexts, thereby limiting the health benefits of increased parental resources [53]. Potential non-linearities across the income distribution also warrant further investigation, particularly to assess whether effects differ in the lower and upper income ranges.

Importantly, immigrant populations are heterogeneous not only in relation to region of origin but also in relation to migration pathways and circumstances. For example, refugees and their descendants may experience different types of barriers than labor migrants or family-reunified migrants [54, 55]. Future research could investigate these factors further.

A different pattern according to sex might have been attributed to different IRs of DUD by sex. Our findings of lower IRs of DUD among females than males were consistent with previous studies; males may have more access to drugs by having a wider social network outside of their home through the labor market and community [17, 37, 56]. In addition, cultural and religious norms for drug use in the original countries may differ [29], and that might have contributed to different patterns by both country of origin and sex. An additional perspective to consider is how differences between immigrants, their descendants, and the majority population may change across generations. While cultural and religious norms related to drug use may differ between countries of origin, such influences may be attenuated in the second generation. Previous research suggests that health behaviors and outcomes in the second generation often converge toward those of the majority population over time, reflecting processes of acculturation and social integration [12]. However, the convergence may vary by country of origin, and persistent disparities may reflect different types of exposures. In this context, the diminished protective effects of parental income observed in our study may indicate that socioeconomic advancement alone may not be sufficient to offset other barriers among certain minority groups [18].

Our findings of the significant association between accumulated exposure to low parental income during childhood and adolescence and DUD in young adulthood were aligned with a previous study, and indicate the importance of prevention strategies in early lifetime [16]. Our study was the first to show that this association was also significantly modified by immigrant background and region of origin, i.e., a weaker association in the second generation was found, especially those from MENA. The weaker association could possibly be a reflection of MDRs due to other risk factors of DUD specific to immigrants. On the other hand, previous studies in Sweden indicate that the risk of psychotic disorders and DUD among immigrants living in neighborhoods with individuals from the same origin would decrease even after controlling for income, by potentially protective effects of ethnic enclaves [45, 57]. As a large proportion of individuals from MENA in Sweden live in the same neighborhoods, social ties and networks attained from the ethnic enclaves might have made the association weaker. However, there is limited evidence of the protective effects of ethnic enclaves on DUD, and such effects were modest [57]. It is important to note that the associations were weaker among individuals from MENA, even though their DUD risk was higher, and they were more socioeconomically disadvantaged than non-immigrants. Future studies can investigate the underlying factors of MDRs for parental income and DUD so that effective policies and interventions can be considered.

Our findings indicated that the age of 10–14 years may be a critical period of exposure to low parental income on DUD risk, which is consistent with a previous study conducted in Denmark [58]. The study reported that exposure to adverse childhood experiences, e.g., parental unemployment during adolescence (i.e., 13–17 years old), was more strongly associated with later age, adverse health outcomes, e.g., mental disorders, than early childhood (i.e., 0–2, 3–5, 6–12 years old). While there is evidence suggesting early childhood is sensitive and critical for neurodevelopment, and often targeted for the period of intervention [59], our findings provide new insight that parental income possibly matters more during this later period for DUD. However, the findings were not as consistent for accumulated exposure, and the pattern was inconsistent across non-immigrants and those with an immigrant background by regions of origin. Future studies could conduct more in-depth analyses to identify critical periods for respective groups.

### Implications

Systematic reviews indicate the effectiveness of income support to households with financial difficulties in reducing the risk of adverse mental health outcomes among children [60, 61]. However, in the presence of MDRs, such policy interventions may not be as effective among immigrant groups, and could sometimes widen the disparity in health between non-immigrants and individuals with immigrant background [18]. Instead, interventions and policies may need to be shaped differently for non-immigrants and second-generation groups, accounting for their regions of origin. Our findings suggest that a large proportion of second-generation individuals, particularly from Africa, Latin America, and MENA, are exposed to low parental income for a longer time period, and their risk of developing DUD was higher. Future studies may need to examine mechanisms behind potential barriers across multiple sectors, including the labor market, schools, and healthcare, working together with state, regions, and municipalities.

### Strengths and limitations

The major strength of our study was the use of whole population data with high-quality information on income, demographic characteristics, and DUD, which made the internal and external validity of the study high. Given that Sweden is among the countries with the highest proportion of immigrant populations, as well as the increasing prevalence of DUD similarly to other countries, our population-level findings may provide insights to countries with a similar context.

There were several methodological limitations in our study. Firstly, our findings were based on data that did not distinguish between specific drug types. The mechanisms in the association between parental income and DUD may differ across drug types, e.g., for cannabis, socioeconomic status may affect initiation and the frequency of use differently; for opioids, socioeconomic status may affect mechanisms, such as those caused by trauma, pain, isolation, and structural disadvantage, differently, and stimulant-related drug problems may arise differently in different social contexts, such as in those related to recreational use [62–64]. Therefore, our findings are generalizable to register-ascertained DUD as a composite outcome, rather than for a single drug category. Future studies could examine cannabis-, opioid-, stimulant-, and polysubstance-related disorders separately. Second, although parental income was assessed before the assessment of DUD, and the individuals with DUD at the age of 15 years were excluded to ensure a temporal relationship, the possibility of reverse causality cannot be ignored. However, our analyses on critical periods of low parental income indicated that parental income assessed in any age period was significantly associated with DUD, and 10–14 years of age was suggested as a more critical period. In addition, our findings were in line with previous studies of parental income and substance use disorders, and, therefore, we believe that the bias induced by the reverse causality was low. On the other hand, excluding the individuals with DUD at the age of 15 years might have biased the results, as the early-onset DUD may still be a consequence of low parental income. Conducting a further sensitivity analysis, including those who already had a DUD record before the age of 15, was however not feasible as DUD cases prior to the age of 15 could not be fully ascertained from certain registers (e.g., the Crime register). Nonetheless, it probably made our analyses conservative, i.e., underestimation of the effect of parental income on DUD, and the excluded individuals were only 2%. Third, to assess the accumulated exposure and critical period of low parental income, we used total household disposable income without equivalization for household size and composition, and defined low income based on a relative threshold. While this approach captures total economic resources in the family, it does not correspond directly with standard at-risk-of-poverty measures based on equivalized income. Fourth, DUD might have been underestimated, even though we assessed DUD by multiple types of registers. However, the underestimation of DUD could have been present for both non-immigrants and second-generation individuals, and, therefore, we believe that this did not bias the associations by immigrant background and region of origin to a high extent. Fifth, the associations between parental income and DUD might have been overestimated, as there are unmeasured confounding factors, even though we adjusted for parental DUD assessed in the lifetime to compensate for this. Sixth, it is possible that DUDs were more likely to be detected in deprived, high-crime neighborhoods, where surveillance by the police may be more extensive.

## Conclusion

Higher parental income was associated with a decreased risk of DUD, and accumulated exposure to low parental income was associated with an increased risk of DUD, while the associations were weaker among second-generation groups from certain regions of origin. These findings suggest that policies aiming to reduce DUD risk among young people may need to address not only financial disadvantage, but also broader social and structural barriers experienced by some populations. Future studies could examine in more detail whether higher financial security in early life may lower the subsequent risk of DUD and why the potentially protective effect is lower among certain second-generation groups.

## Supplementary Information

Supplementary Material 1.

## Data Availability

The datasets presented in this article are not readily available due to legal concerns. Further information regarding the register data can be found in the Swedish National Board of Health and Welfare ( [https://www.socialstyrelsen.se/en/statistics-and-data/registers/](https:/www.socialstyrelsen.se/en/statistics-and-data/registers) ).
