# Supplementary material for: Parental income and drug use disorder among second-generation individuals in Sweden: effect modification by immigrant background and region of origin
Source: BMC Public Health. 2026 May 29;26:1745. doi: 10.1186/s12889-026-27931-y (PMC13220473; doi:10.1186/s12889-026-27931-y)
Supplement: Supplementary file 1 — Supplementary Material 1. [file 12889_2026_27931_MOESM1_ESM.docx]

**Supplementary appendix**

[Table 1: Swedish register data used in the study 2](#_Toc229501823)

[Table 2: Definitions of drug use disorder by register and its identification codes that are used in this study 3](#_Toc229501824)

[Table 3: The list of countries by region of origin for second-generation individuals. 4](#_Toc229501825)

[Table 4: Association between parental income and drug use disorder according to immigrant background 5](#_Toc229501826)

[Table 5: Association between parental income and drug use disorders according to immigrant background and region of origin 6](#_Toc229501827)

[Figure 1: Flow chart of the study population 7](#_Toc229501828)

[Figure 2: Proportion of parents receiving social welfare assistance according to parental income in 100 percentiles 8](#_Toc229501829)

[Figure 3: Hazard ratio (HR) of drug use disorder by accumulated exposure to parents receiving social welfare assistance according to immigrant background and region of origin. 9](#_Toc229501830)

[Figure 4: Association between specific period of low parental income and drug use disorders according to immigrant background and region of origin – to assess critical periods 10](#_Toc229501831)

# **Table 1: Swedish register data used in the study**

| **Data** | **Available year of data and Description** | **Assessed variables** |
| --- | --- | --- |
| Multi-Generation Register | 1932-2020.  Linked children and parents who were born in Sweden or immigrated to Sweden together with parents after 1932. | Immigrant background.  Region of origin. |
| Total Population Register | 1968-2020.  Sociodemographic information. | Sex.  Age.  Immigrant background.  Region of origin.  Emigration. |
| National Patient Register |  |  |
| Inpatient records | 1964-2020.  Hospitalization. | Drug use disorder.  Parental drug use disorder. |
| Outpatient records | 2001-2020.  Hospital visits. | Drug use disorder.  Parental drug use disorder. |
| Crime Register | 1973-2020.  Convictions in lower courts of law. Suspect of a crime. | Drug use disorder.  Parental drug use disorder. |
| Longitudinal Integration Database for Health Insurance and Labor Market Studies (LISA) | 1990-2020.  Socioeconomic information. | Parental income.  Parental education.  Parental social welfare assistance. |
| Cause of Death Register | 1961-2020.  Information of all deaths. | To censor individuals. |

# **Table 2: Definitions of drug use disorder by register and its identification codes that are used in this study**

| National Patient Register: Inpatient and Outpatient records | |
| --- | --- |
| *ICD-9* | |
| Drug psychoses. | 292 |
| Drug dependence. | 304 |
| Cannabis abuse, Hallucinogens abuse (Include: LSD reaction), Barbiturates and tranquillizers abuse, Morphine type abuse, Cocaine type abuse, Amphetamine type abuse, Antidepressants abuse, Other, mixed or unspecified abuse of drugs. | 305C-305I |
| *ICD-10* | |
| Opioid related disorders, Cannabis related disorders, Sedative, hypnotic, or anxiolytic related disorders, Cocaine related disorders, Other stimulant related disorders, Hallucinogen related disorders, Inhalant related disorders, Other psychoactive substance related disorders. | F11-F16 and F18-F19 |
| Crime Register | |
| *Suspicion and Convictions in lower courts* | |
| Driving under the influence of narcotics. | 3070 |
| Use of narcotics. | 5011 |
| Possession^1^ of narcotics. | 5010 |
| Use and possession^1^ of narcotics. | 5012 (available 2000 - 2007) |
| Use and/or possession^1^ of narcotics. | Law 1968:64, paragraph 1, point 6^2^ |
| Driving under the influence of narcotics. | Law 1951:649, paragraph 4, subsection 2 and paragraph 4A, subsection 2 |

^1^Possession of narcotics was assessed whether that indicates use. Specifically, over 90% of individuals suspected of possession had at least one other registration for drug use disorders in the Inpatient, Outpatient, Crime, or Suspicion register.

^2^In 2023, the Swedish Penal Law on Narcotics was amended, reducing the number of points from six to five by moving point 6 to point 5.

# **Table 3: The list of countries by region of origin for second-generation individuals.**

| Africa | Angola, Benin, Botswana, Burkina Faso, Burundi, Cameroon, Cape Verde, Central African Republic, Chad, Comoros, Congo (Republic of the), Congo (Democratic Republic of the), Côte d'Ivoire, Djibouti, Equatorial Guinea, Eritrea, Ethiopia, Gabon, Ghana, Guinea, Guinea-Bissau, Kenya, Lesotho, Liberia, Madagascar, Malawi, Mali, Mauritius, Mozambique, Namibia, Niger, Nigeria, Rwanda, São Tomé and Príncipe, Senegal, Seychelles, Sierra Leone, Somalia, Sudan, South Sudan, South Africa, Swaziland, Tanzania, The Gambia, Togo, Uganda, Zambia, Zanzibar (Tanzania), Zimbabwe |
| --- | --- |
| Asia and Oceania | Afghanistan, Bangladesh, Bhutan, Brunei Darussalam, Cambodia, China, Philippines, Hong Kong (China), India, Indonesia, Japan, Kazakhstan, Kyrgyzstan, South Korea, North Korea, Laos, Malaysia, Maldives, Mongolia, Myanmar, Nepal, Pakistan, Sikkim (India), Singapore, Sri Lanka, Tajikistan, Taiwan, Thailand, Turkey, Turkmenistan, Uzbekistan, Vietnam, East Timor, Fiji, Kiribati, Micronesia, Nauru, Papua New Guinea, Solomon Islands, Samoa, Tonga, Vanuatu |
| Eastern Europe | Albania, Armenia, Azerbaijan, Belarus, Bosnia-Herzegovina, Bulgaria, Czech Republic, Estonia, Georgia, Hungary, Kosovo, Croatia, Latvia, Lithuania, North Macedonia, Moldova, Montenegro, Poland, Romania, Serbia, Slovakia, Slovenia |
| Latin America and the Caribbean | Antigua and Barbuda, Argentina, Belize, Bahamas, Barbados, Bermuda (UK), Bolivia, Brazil, Chile, Colombia, Costa Rica, Cuba, Dominican Republic, Ecuador, El Salvador, Grenada, Guatemala, Guyana, Haiti, Honduras, Jamaica, Virgin Islands (US), Mexico, Nicaragua, Panama, Paraguay, Peru, St Lucia, St Vincent and the Grenadines, Surinam, Trinidad and Tobago, Uruguay, Venezuela |
| Middle East and North Africa | Algeria, Bahrain, Egypt, Iraq, Iran, Jordan, Kuwait, Lebanon, Libya, Morocco, Mauritania, Oman, Palestine, Qatar, Saudi Arabia, Syria, Tunisia, UAE, Yemen |
| West | Andorra, Australia, Austria, Belgium, Canada, Cyprus, Denmark, Gibraltar (UK), Greece, Finland, France, Iceland, Ireland, Israel, Italy, Liechtenstein, Luxembourg, Malta, Monaco, Netherlands, Norway, New Zealand, Portugal, San Marino, Switzerland, Spain, UK, Germany, USA |

**Notes:** Regions were categorized according to geographic, cultural, and economic similarities, which were used in previous studies.

# **Table 4: Association between parental income and drug use disorder according to immigrant background**

|  | **Non-immigrants** |  | **Second generation** | |
| --- | --- | --- | --- | --- |
|  | **Model 1** | **Model 2** | **Model 1** | **Model 2** |
|  | **HR (95%CI)** | **HR (95%CI)** | **HR (95%CI)** | **HR (95%CI)** |
| **Males** |  |  |  |  |
| **Parental income** |  |  |  |  |
| By ten percentile | 0.85 (0.84; 0.85) | 0.88 (0.88; 0.89) | 0.87 (0.86; 0.87) | 0.90 (0.90; 0.91) |
| **Females** |  |  |  |  |
| **Parental income** |  |  |  |  |
| By ten percentile | 0.80 (0.79; 0.80) | 0.84 (0.83; 0.84) | 0.83 (0.82; 0.84) | 0.87 (0.86; 0.89) |

Model 1: Crude model. Model 2: Adjusted for birth year, parental education, and parental drug use disorder.

# **Table 5: Association between parental income and drug use disorders according to immigrant background and region of origin**

|  | **Non-immigrants** | | **Second generation** | | | | | |
| --- | --- | --- | --- | --- | --- | --- | --- | --- |
|  | **Sweden** | **Africa** | | **Asia** | **East Europe** | **Latin America** | **MENA** | **West** |
|  | **HR**  **(95%CI)** | **HR**  **(95%CI)** | | **HR**  **(95%CI)** | **HR**  **(95%CI)** | **HR**  **(95%CI)** | **HR**  **(95%CI)** | **HR**  **(95%CI)** |
| **Males** |  |  | |  |  |  |  |  |
| **Model 1** |  |  | |  |  |  |  |  |
| **Parental income** | | | | | | | | |
| By ten percentile | 0.85  (0.84; 0.85) | 0.89  (0.87; 0.91 | | 0.91  (0.89; 0.93) | 0.86  (0.85; 0.88) | 0.88  (0.86; 0.90) | 0.90  (0.88; 0.91) | 0.86  (0.85; 0.87) |
| **Model 2** |  |  | |  |  |  |  |  |
| **Parental income** | | | | | | | | |
| By ten percentile | 0.88  (0.88; 0.88) | 0.93  (0.91; 0.95) | | 0.93  (0.92; 0.95) | 0.90  (0.89; 0.91) | 0.92  (0.90; 0.94) | 0.92  (0.91; 0.94) | 0.90  (0.89; 0.91) |
| **Females** |  |  | |  |  |  |  |  |
| **Model 1** |  |  | |  |  |  |  |  |
| **Parental income** | | | | | | | | |
| By ten percentile | 0.80  (0.79; 0.80) | 0.85  (0.80; 0.88) | | 0.84  (0.81; 0.87) | 0.83  (0.80; 0.85) | 0.86  (0.83; 0.90) | 0.85  (0.82; 0.87) | 0.81  (0.80; 0.83) |
| **Model 2** |  |  | |  |  |  |  |  |
| **Parental income** | | | | | | | | |
| By ten percentile | 0.84  (0.83; 0.84) | 0.89  (0.85; 0.93) | | 0.87  (0.84; 0.90) | 0.87  (0.84; 0.89) | 0.90  (0.87; 0.94) | 0.88  (0.86; 0.91) | 0.86  (0.84; 0.87) |

Model 1: Crude model. Model 2: Adjusted for birth year, parental education, and parental drug use disorder. Latin America: Latin America or Caribbean. MENA: Middle East or North Africa.

# **Figure 1: Flow chart of the study population**


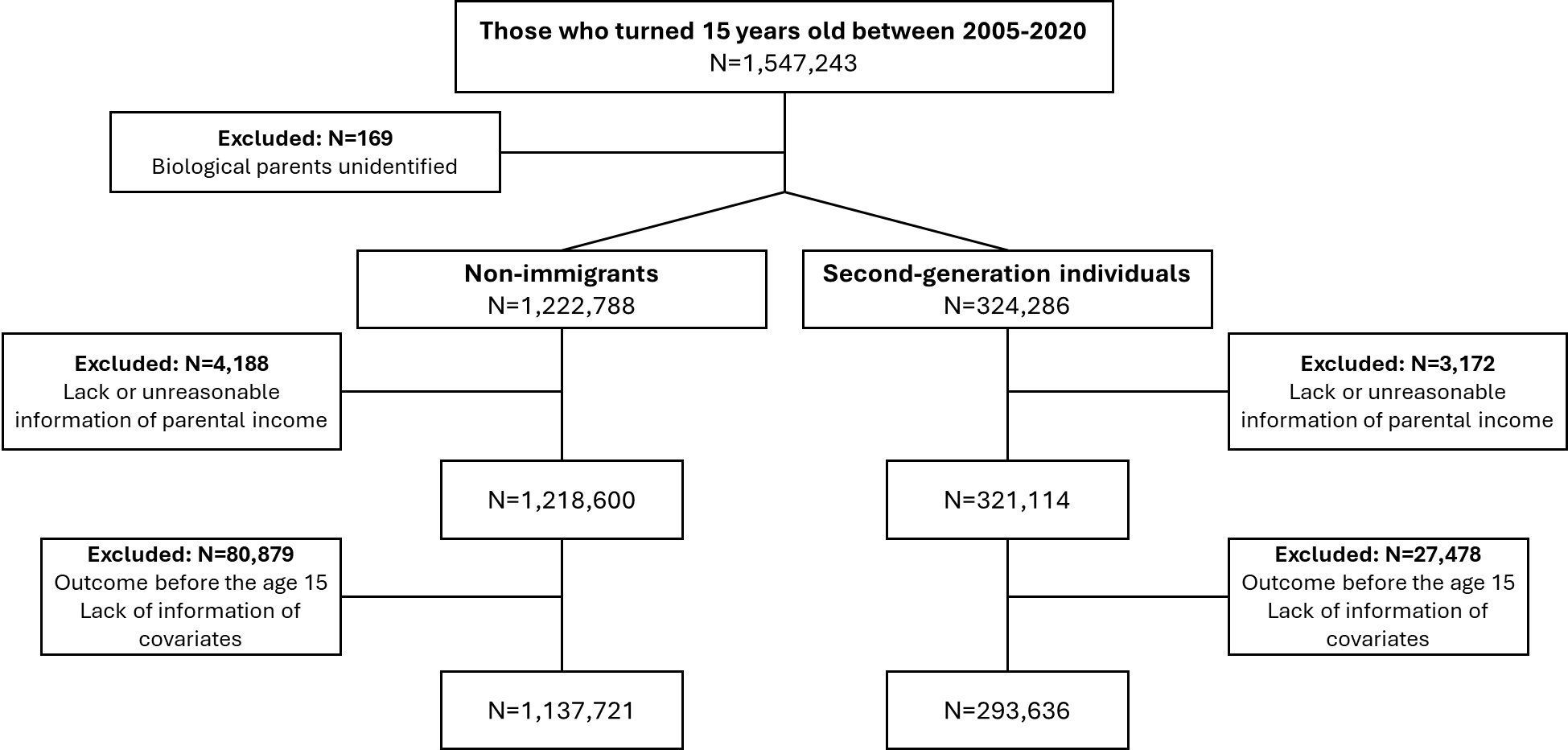


# **Figure 2: Proportion of parents receiving social welfare assistance according to parental income in 100 percentiles**


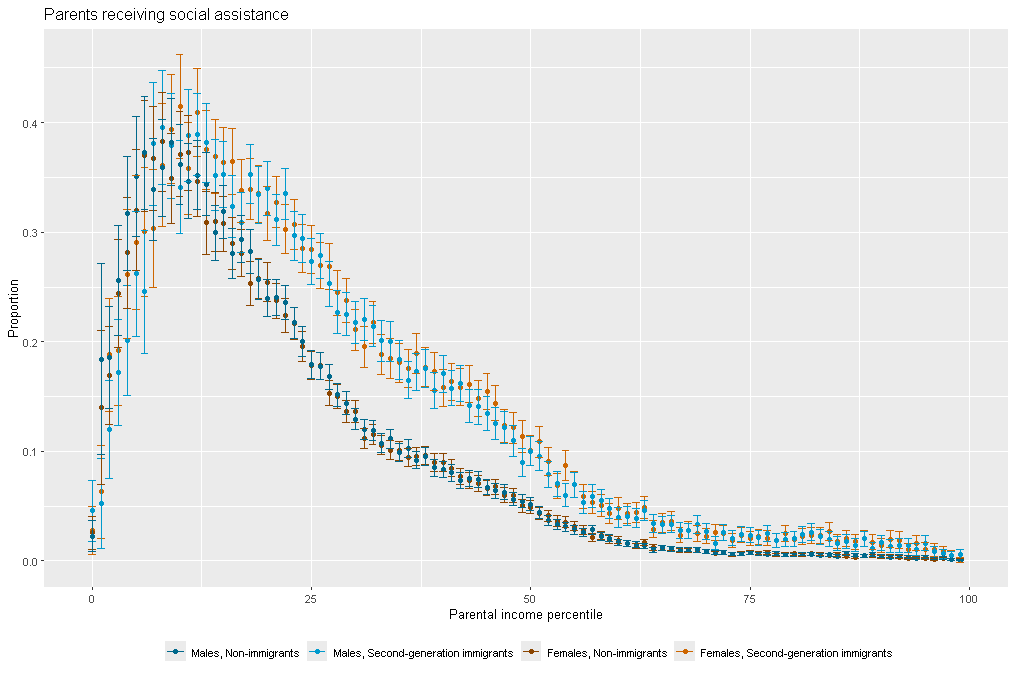


**Footnotes:** Both social welfare assistance receiving status and parental income were assessed when the study individuals were at the age of 14.

# **Figure 3: Hazard ratio (HR) of drug use disorder by accumulated exposure to parents receiving social welfare assistance according to immigrant background and region of origin.**

**
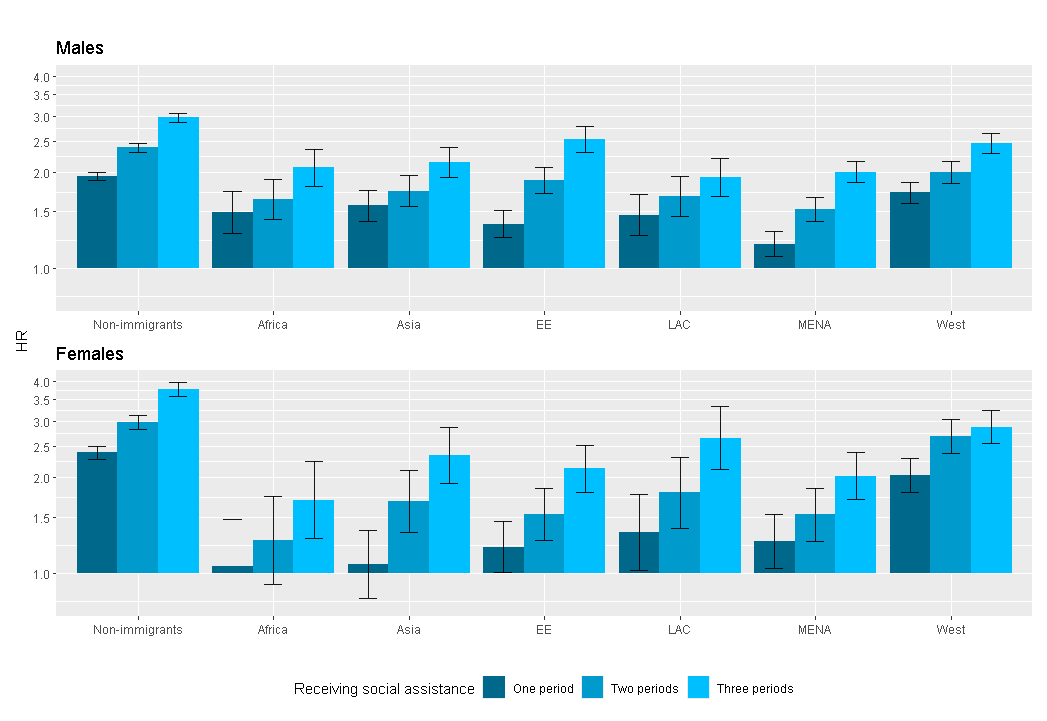
**

**Footnotes:** HRs were derived from the Cox proportional hazard models adjusting for birth year, parental education, and parental drug use disorders. Y-axis is in log-scale. Not exposed to any periods is the reference. EE: East Europe. LAC: Latin America or Caribbean. MENA: Middle East or North Africa.

# **Figure 4: Association between specific period of low parental income and drug use disorders according to immigrant background and region of origin – to assess critical periods**

**
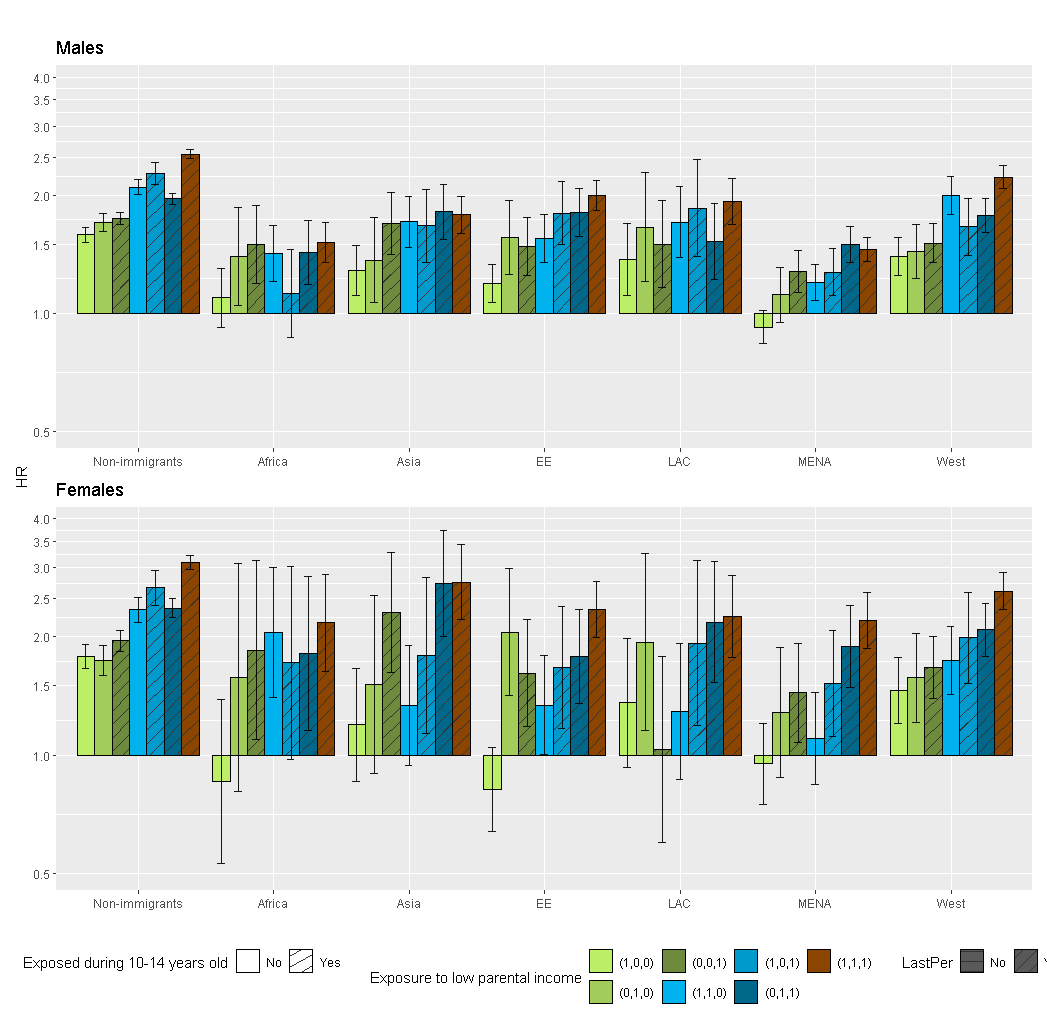
**

Adjusted for birth year, parental education, and parental drug use disorder. EE: East Europe. LAC: Latin America or Caribbean. MENA: Middle East or North Africa. Exposure to low parental income: 1=exposed, 0=not exposed for the age periods (0-4, 5-9, 10-14).
